# Comprehensive geriatric assessment in older patients with cancer: an external validation of the multidimensional prognostic index in a French prospective cohort study

**DOI:** 10.1186/s12877-020-01692-8

**Published:** 2020-08-18

**Authors:** Evelyne Liuu, Chunyun Hu, Simon Valero, Thomas Brunet, Amelie Jamet, Marie-Laure Bureau, Alberto Pilotto, Pierre-Jean Saulnier, Marc Paccalin

**Affiliations:** 1 Department of Geriatrics, Poitiers University Hospital, Poitiers, France; 2 Clinical Investigation Centre CIC1402, CHU Poitiers, University of Poitiers, INSERM, Poitiers, France; 3 Department Geriatric Care, Orthogeriatrics and Rehabilitation, Frailty Area, E.O. Galliera Hospital, Genova, Italy; 4 Department of interdisciplinary Medicine, Aldo Moro University of Bari, Bari, Italy

**Keywords:** Aged, Neoplasms, Mortality, Comprehensive geriatric assessment

## Abstract

**Background:**

Older patients with cancer require specific and individualized management. The 3-group Multidimensional Prognostic Index (MPI) based on the Comprehensive Geriatric Assessment (CGA) has shown a predictive interest in terms of mortality. The objective of our study was to assess the prognostic value of MPI for 1-year mortality in an external prospective French cohort of elderly patients with cancer.

**Methods:**

From March 2015 to March 2017 a prospective single-center cohort study enrolled all patients with cancer, aged 75 years and older referred to the geriatric oncology clinic. We used a proportional hazard model for 1-year mortality adjusted for age, sex, tumor sites and metastatic status. C-statistics were used to assess the incremental predictive value of MPI index to these risk factors.

**Results:**

overall, 433 patients underwent CGA with MPI (women 42%; mean age 82.8 ± 4.8 years). The most common tumor sites were prostate (23%), skin (17%), colorectum (15%) and breast (12%); 29% of patients had a metastatic disease; 231 patients (53%) belonged to the “MPI-1” group, 172 (40%) to the “MPI-2” group and 30 patients were classified in the “MPI-3” group. One-year mortality rate was 32% (23% in MPI-1, 41% in MPI-2 and 53% in MPI-3, *p* = 0.024). All domains of MPI except cognition and living status were significantly associated with mortality at one-year, as well as tumor sites and metastatic status. Higher MPI was associated with a higher mortality risk (adjusted HR 1.56 [95%CI 1.70–2.09] and 1.72 [1.33–2.22] for MPI groups 2 and 3 compared to 1; *p* < 0.0001).

**Conclusions:**

In addition to established risk factors, MPI improves risk prediction of 1-year mortality. This practical prognostic tool may help to optimize management of these vulnerable patients.

## Background

Individuals over 65 years old are the fastest growing segment of the population, and by 2030 will represent about 20% of Americans and 25% of Europeans [1]. The incidence of cancer continues to increase worldwide: it is estimated at 23.6 million/year by 2030, representing an increase of 68% in cases compared with 2012 [2]. The incidence of cancer is 11 times higher in people over 65 years old, and people aged 70 and older have a higher risk of developing invasive cancer [3].

The older population is characterized by a very heterogeneous profile, especially in terms of frailty, geriatric characteristics, and comorbidities, which explains the need for specific and adapted care [4, 5]. Nevertheless, scientific data are scarce because older subjects are often under-represented in oncological clinical trials that set the standards of antineoplastic treatment [6, 7].

Over the last three decades, the five-year survival rate for all types of cancer has increased, particularly in individuals aged 50 to 64 [3, 8]. Still, older patients are at more risk of toxicity in anti-cancer therapies such as chemotherapy, and require a benefit/risk assessment prior to treatment [4]. A comprehensive geriatric assessment (CGA) is consequently recommended in these patients to diagnose comorbidities and optimize geriatric interventions, and to improve the functional state and possibly the survival rate, by ensuring better tolerance to treatment [9, 10]. CGA has also shown predictive value in identifying elderly patients with cancer who are exposed to a poor prognosis, including a higher risk of death during hospitalization [11]. Among the CGA-based assessment tools, the Multidimensional Prognostic Index (MPI) has shown a predictive interest in mortality at 6 months and 12 months in Italian patients aged 70 years and older with advanced cancers [12–15].

The main objective of our study was to validate the prognostic value of the MPI for 1-year mortality in an external French cohort of older patients with cancer. The secondary objective was to assess the major risk factors associated with 12-month mortality in these patients.

## Methods

### Study population and data collection

This prospective single-center cohort study enrolled from March 2015 to March 2017 all patients with cancer, aged 75 years and older, who were referred to the geriatric oncology clinic of Poitiers University Hospital, prior to planned anti-cancer treatment. Socio-demographic data and cancer-related information were collected during the consultation, including age, sex, marital status, social environment, type of cancer, metastasis status, and cancer-specific treatment. Tumor sites were classified as follows: colorectal, breast, prostate, upper gastrointestinal tract (stomach and esophagus) and liver, urinary system (bladder, upper urinary tract, and kidney), hematologic malignancies, and other tumors (including ovary, uterus, lung, head and neck, skin, thyroid, and unknown primary). The CGA was performed by a senior geriatrician specialized in oncology and provided data necessary to calculate MPI. All eligible patients who had signed the consent form were included in the study. The study protocol was validated by the Poitiers University Hospital ethics committee, Poitiers, France. All the clinical and biological data were collected and recorded in a cohort database.

### Multidimensional prognostic index

The MPI, based on a CGA, was calculated after administration of standardized and validated tests exploring eight domains (Table 1) [14]. Living status was categorized as “living with family”, “institutionalized” or “alone”, and functional status was evaluated by Activities of Daily Living (ADL) ranging from 0 (total dependence) to 6 (independence) and Instrumental ADL (IADL) [16, 17]. Nutrition was assessed by the Mini Nutritional Assessment-Short Form (MNA-SF) questionnaire; cognitive status was evaluated by the Short Portable Mental Status Questionnaire (SPMSQ) [18, 19]. The Exton-Smith Scale (ESS) estimated the risk of pressure ulcer [20]. Comorbidities were evaluated by the Cumulative Illness Rating Scale (CIRS), which scores the severity of 14 organic systems, ranging from 0 (absent) to 4 (most severe) [21]. Based on this scale, a comorbidity index (CIRS-CI) records the number of moderate to severe organ pathologies (CIRS scores from 2 to 4) [22]. The number of medications is classified in three groups: “≤3 drugs a day”, “4 to 6 drugs” or “≥7 drugs”.

**Table 1:** Multidimensional Prognostic Index score assigned to each domain according to the severity of problem

Assessment tests (range)	No problem (value = 0)	Minor problem (value = 0.5)	Severe problem (value = 1)
ADL (0–6)	≥5	4–3	≤2
IADL (0–8)	≥6	5–4	≤3
SPMSQ (0–10)^a^	≤3	4–7	≥8
CIRS-CI (0–14)^b^	0	1–2	≥ 3
MNA-SF (0–17)	≥ 12	8–11	≤ 7
ESS (5–25)	≥16	10–15	5–9
Number of medications	0–3	4–6	≥ 7
Living status	Living with family	Institutionalized	Living alone

*Abbreviations: ADL* Activities of Daily Living, *IADL* Instrumental Activities of Daily Living, *SPMSQ* Short Portable Mental Status Questionnaire, *CIRS-CI* Cumulative Illness Rating Scale Comorbidity Index, *MNA-SF* Mini Nutritional Assessment Short Form, *ESS* Exton Smith Scale

^a^ Number of errors

^b^ Number of pathologies

The MPI was scored by matching the results of these tests. A value of “0”, “0.5” or “1” was assigned according to the conventional cutoff points, considering “0” as no problem, “0.5” minor problem and “1” major problem (Table 1). The sum was then divided by 8 to obtain the final MPI score, which was categorized into 3 groups: the “MPI-1” group (final score ≤ 0.33, defining patients with low mortality risk at 1 year), the “MPI-2” group (0.34–0.66, moderate risk) and the “MPI-3” group (group > 0.66, higher risk).

### Definition of outcomes

The primary outcome in the longitudinal analyses was 1-year mortality. Systematic follow-up was performed after discharge through clinical visits every 6 months by the same clinical research assistant. When patients were not present at visit, phone calls were made to the general practitioners to assess vital status and to obtain the date of death if applicable.

### Statistical analysis

Descriptive statistics were reported as mean ± standard deviation (SD) or median (25th–75th percentiles) for continuous variables or absolute number and percentage for categorical variables. The time to event was plotted as Kaplan-Meier survival curves according to MPI groups, and comparison was made using the log-rank test. The hazard ratio (HR) of 1-year mortality for each parameter was determined by Cox proportional hazards regression. Two models were used: univariate model, and models adjusted for age, sex, metastatic status, tumor sites. Interactions between sex, tumor site and metastatic status for the association between MPI and 1-year mortality were evaluated by the addition of interaction terms into the corresponding regression model. The Akaike’s information criterion (AIC) was used to compare global fit among models (with and without MPI), and the model with the smallest AIC was considered as the best model.

Generalized c-statistics were calculated to assess improvement in 1-year mortality risk prediction of MPI in addition to traditional risk factors: age, sex, metastatic status, tumor sites [23]. The 95% CIs for the changes in the c-statistic were computed based on 1000 bootstrap samples. *P* values < 0.05 were considered statistically significant. Statistical analyses were performed with SAS version 9.4 (SAS Institute, Cary, NC).

## Results

### Baseline characteristics of study population

During the recruitment period, 433 eligible patients aged 75 years and older were included, mostly males (*n* = 252, 58%), with a mean age of 82.8 ± 4.8 years (Table 2). The most common tumor sites were prostate (23%), skin (17%) and breast (12%); 29% of patients had a metastatic disease. Anti-cancer treatment included chemotherapy in 162 patients (37%), surgery in 137 (32%) and radiotherapy in 109 (25%). Patients had comorbid conditions regarding the CIRS-scale and medication and were frequently malnourished (29%) (Table 2). In this cohort, 231 patients (53%) were classified in the “MPI-1” group, 172 patients (40%) in “MPI-2” and 30 patients in “MPI-3”. Except for metastatic status and antineoplastic treatments, all variables of interest differed between the three MPI groups (*P* ≤ 0.02).

**Table 2:** Patients’ baseline characteristics and evaluation by multidimensional prognostic index MPI (*n* = 433)

		Total cohort	MPI-1	MPI-2	MPI-3	P
		*N* = 433	*N* = 231	*N* = 172	*N* = 30	
**Sociodemographic characteristics**						
Age		82.8 ± 4.8	82.1 ± 4.5	83.5 ± 5.0	83.9 ± 5.7	0.007
Female n (%)		181 (42%)	82 (35%)	83 (48%)	16 (53%)	0.02
**Oncological characteristics**						
Most frequent tumor sites						0.02
	Prostate	98 (23%)	75 (32%)	20 (12%)	3 (10%)	
	Skin	72 (17%)	38 (16%)	26 (15%)	8 (3%)	
	Colorectum	66 (15%)	34 (15%)	28 (16%)	4 (13%)	
	Breast	52 (12%)	26 (11%)	23 (13%)	3 (10%)	
	Hematological malignancies	37 (9%)	12 (5%)	24 (14%)	4 (13%)	
	Bladder	34 (8%)	17 (7%)	14 (8%)	3 (10%)	
Metastatic status n (%)		125 (29%)	70 (30%)	48 (28%)	7 (23%)	0.70
Type of antineoplastic treatment ^a^						0.07
	Chemotherapy	162 (37%)	80 (35%)	72 (42%)	10 (33%)	
	Surgery	137 (32%)	73 (32%)	48 (28%)	16 (53%)	
	Radiotherapy	109 (25%)	72 (31%)	35 (20%)	2 (7%)	
	Hormone therapy	32 (7%)	24 (10%)	8 (5%)	0	
**Comprehensive geriatric assessment and multidimensional prognostic index** ^**b**^						
ADL score		5.2 ± 1.3	5.7 ± 0.6	5.1 ± 1.2	2.2 ± 1.4	< 0.0001
ADL category		364/42/27	226/4/1	135/29/8	3/9/18	< 0.0001
IADL score		5.1 ± 2.6	6.4 ± 1.7	4.2 ± 2.5	0.9 ± 1.0	< 0.0001
IADL category		221/95/117	165/54/12	56/41/75	0/0/30	< 0.0001
ESS score		17.8 ± 2.4	18.9 ± 1.4	17.1 ± 2.4	13.1 ± 2.3	< 0.0001
ESS category		330/62/41	198/4/29	129/33/10	3/25/2	< 0.0001
MNA-SF score		9.3 ± 2.9	10.5 ± 2.4	8.1 ± 2.7	6.0 ± 2.1	< 0.0001
MNA-SF category		102/204/127	90/117/24	11/83/78	1/4/25	< 0.0001
SPMSQ score		1.6 ± 2.0	1.1 ± 1.4	1.8 ± 2.1	4.5 ± 3.0	< 0.0001
SPMSQ category		372/44/9	220/7/1	141/25/3	11/12/7	< 0.0001
CIRS score		7.4 ± 4.2	6.2 ± 3.5	8.3 ± 3.8	12.2 ± 6.0	< 0.0001
CIRS-CI score		2.8 ± 1.7	2.2 ± 1.5	3.1 ± 1.5	4.5 ± 2.4	< 0.0001
CIRS-CI category		21/196/216	19/132/80	2/58/112	0/6/24	< 0.0001
Number of medications		6.7 ± 3.1	5.8 ± 3.0	7.6 ± 2.7	9.2 ± 3.2	< 0.0001
Number of medications category		55/162/215	48/107/76	7/50/114	0/5/25	< 0.0001
Living status (family/institution/alone)		263/43/115	186/5/33	71/23/73	6/15/9	< 0.0001
MPI score		0.393 ± 0.394	0.252 ± 0.225	0.494 ± 0.337	0.902 ± 0.871	< 0.0001

Numbers are mean ± SD or n (%)

*Abbreviations: MPI* multidimensional prognostic index, *SD* standard deviation, *ADL* activities of daily livings, *IADL* instrumental activities of daily livings, *ESS* Exton-Smith Scale, *MNA-SF* mini nutritional assessment short form, *SPSMQ* Short Portable Mental Status Questionnaire, *CIRS* Cumulative Illness Rating Scale, *CI* comorbidity index

^a^ Antineoplastic treatment may combine one or several types of treatment

^b^ Categories are reported as number of patients with no/minor/severe problem to calculate MPI score

### MPI and 1-year mortality

**Fig. 1:**
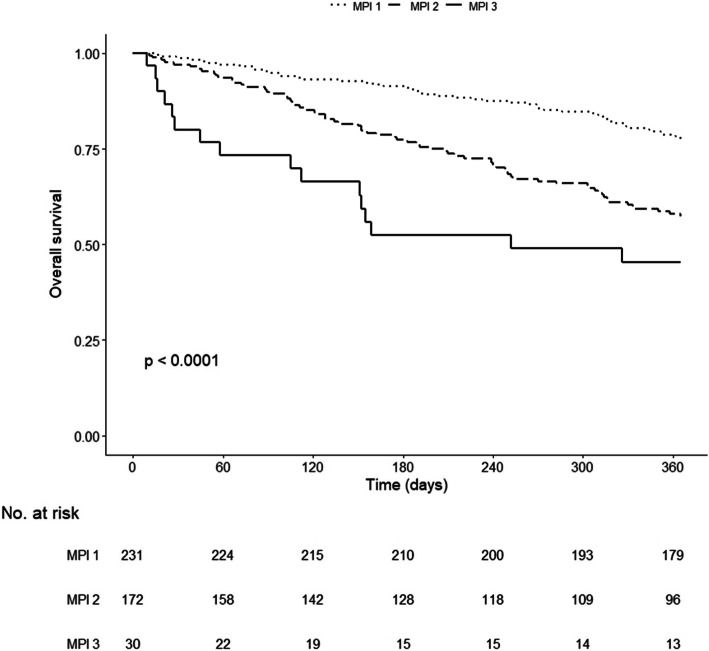
Kaplan-Meier curves of overall mortality in 433 patients according to MPI groups. Dotted line, MPI 1; dashed line, MPI 2 and solid line, MPI 3. Log-rank test *P* < 0.0001

Among the 433 patients, 12 were lost to follow-up (3%). Mean follow-up was 13.7 ± 6.4 months. Overall mortality at 12 months was 32% (23% in MPI-1, 41% in MPI-2 and 53% in MPI-3, *P* = 0.02) (Fig. 1).

Since we observed significant statistical interaction between sex and tumor site (*P* = 0.01), we presented results for the multivariate model with the inclusion in the model of an interaction term. We found no significant interaction between tumor site and metastatic status (*P =* 0.98).

The risk of 1-year mortality across MPI groups is shown in Fig. 1.

All functional scoring but SPMSQ and living status, number of daily drugs, metastatic status and tumor site were significantly associated with mortality (Table 3). Compared to colorectal cancer (reference category), breast cancer was associated with significantly lower 1-year mortality and upper gastrointestinal tract/liver cancer and other malignancies with significantly higher 1-year mortality.

**Table 3:** Univariate and multivariate analyses for one-year mortality, model for MPI-groups (*n* = 433)

Variable	HR (95% CI)	***P***-value	Adjusted HR (95% CI)	P
ADL score (+ 1 point)	0.82 (0.74–0.91)	0.0003	–	
IADL score (+ 1 point)	0.85 (0.80–0.90)	< 0.0001	–	
SPMSQ score (+ 1 point)	1.00 (0.92–1.09)	0.9577	–	
CIRS score (+ 1 point)	1.09 (1.05–1.13)	< 0.0001	–	
MNA score (+ 1 point)	0.78 (0.73–0.82)	< 0.0001	–	
ESS score (+ 1 point)	0.88 (0.84–0.93)	< 0.0001	–	
Number of drugs (+ 1 drug)	1.05 (1.00–1.11)	0.0463	–	
Living status			–	
living with family	reference	0.6817		
living alone	1.02 (0.69–1.51)			
Institutionalized	1.27 (0.74–2.17)			
Age (+ 1 year)	1.01 (0.98–1.05)	0.4937	0.99 (0.95–1.02)	0.5149
Sex (male vs female)	0.95 (0.68–1.33)	0.7500	0.18 (0.06–0.57)	0.0035
Metastatic status	2.01 (1.43–2.81)	<.0001	2.46 (1.72–3.53)	<.0001
Tumor sites				
Colorectal	reference	< 0.0001	reference	< 0.0001
breast	0.79 (0.37–1.71)		0.14 (0.05–0.38)	
prostate	0.27 (0.11–0.67)		0.59 (0.21–1.65)	
Upper gastrointestinal tract/liver	0.18 (0.08–0.41)		0.75 (0.37–1.52)	
urinary system	1.43 (0.55–3.73)		0.42 (0.18–1)	
hematologic malignancies	1.15 (0.61–2.16)		0.46 (0.21–0.99)	
other tumors	1.1 (0.62–1.97)		0.23 (0.1–0.55)	
Multidimensional Prognostic Index				
group 1	reference	< 0.0001	reference	< 0.0001
group 2	2.19 (1.53–3.14)		2.06 (1.42–2.98)	
group 3	3.65 (2.08–6.4)		4.34 (2.41–7.82)	

*Abbreviations: HR* hazard ratio, *CI* confidence interval, *MPI* Multidimensional Prognostic Index

Multivariate model adjusted for age, sex, tumor site, metastatic status and MPI groups

MPI groups were associated with 1-year mortality in the univariate model and remained significantly associated even after adjustment for age, sex, metastatic status and tumor site. Compared to the MPI-1 group, patients of the MPI-2 and MPI-3 groups had gradual increased risk of 1-year mortality (adjusted hazard ratio [95%CI], 2.06 [1.42–2.98] and 4.34 [2.41–7.82], respectively, *P* < 0.0001) (Table 3).

### Discrimination

We assessed improvement in risk discrimination for the MPI group compared with the model with traditional risk factors (age, sex, metastatic status and tumor site). We observed a small but significant improvement in 1-year mortality risk prediction (difference in C-statistic = 0.027, *P*= 0.001), when including the MPI group in the model (Table 4).

**Table 4:** Predictive performance of MPI during 12-month follow-up

Biomarker	Akaike criterion	c-index	(95% CI)	difference in C-statistics	P value
clinical model	1589.1	0.681	(0.638–0.723)		
clinical model +MPI	1563.0	0.708	(0.667–0.748)	0.027	0.001

Clinical model: age, sex, metastatic status, tumor site

## Discussion and implications

Our study confirmed the predictive value of the multidimensional prognostic index for 1-year mortality in older patients with cancer. MPI group 3 had a significantly two- to five-fold higher rate of 1-year mortality. We also showed that the MPI improved prediction of 1-year mortality, going beyond the traditional risk factors reported in the literature [24].

Estimation of patient survival at time of the therapeutic decision is required to assess the balance of benefits and risks of performing or not performing specific oncologic interventions, taking cancer-specific mortality into consideration. Clinicians may need to know if the patient will die of cancer or with cancer, in cases where comorbidities or geriatric syndromes are challenging. Several scales have been created and validated in large epidemiologic cohorts to estimate overall survival, notably at 12 months with the Carey and Walter indexes [5, 25, 26]. These two scores consider dependency, comorbidities with cancer, and malnutrition. Walter and collaborators reported independent associations between one-year mortality in multivariable analysis and risk factors, including male gender, two medical diagnoses: congestive heart failure (aOR 1.4 (95%CI 1.1–1.8)), and cancer (aOR 2.6 (1.7–3.9)) for localized cancer and aOR 13.4 (6.2–29.0), for metastatic cancer), functional dependency in any ADL at discharge (aOR 2.1 (1.6–2.8), for dependencies from 1 to 4 ADLs, and aOR 5.7 (4.2–7.7), for dependencies in all ADLs), and 2 laboratory values: creatinine level > 3.0 mg/dL [265.2 μmol/L], aOR 1.7 (1.2–2.3)) and albumin level ≤ 3.4 g/dL, aOR 1.7 (1.2–2.3), from 3.0 to 3.4 g/dL and aOR 2.1 (1.4–3.0), for values below 3.0 g/dL) [5]. Carey et al. confirmed these findings and furthered the elaboration of a prognostic index for mortality in community-living frail older individuals, considering eight independent risk factors of mortality, weighted using Cox regression: male sex, dependence in toileting, malignant neoplasm, and renal insufficiency [25]. None of these tests were specifically developed in cohorts with individuals with cancer, and they may consequently not be informative enough to reflect clinical and functional variability in daily care and to provide personalized corrective interventions. Recent evidence reported a positive impact of geriatric interventions and monitoring in survival increase, improvement of quality of life, and completion of chemotherapy [27, 28]. The MPI differs from other mortality indexes because it is based on a CGA, with each of the eight tests assessing one geriatric domain. Giantin and collaborators confirmed the good discriminatory power for 12-month mortality in a cohort of 160 cancer patients older than 70, and validated higher mortality prediction compared to a standard CGA [13]. Use of the MPI in clinical practice may provide rapid and comprehensive evaluation of patients, and help to adapt decision-making in oncology.

The MPI has been developed and validated in large cohorts of in and outpatients for many causes, to predict not only mortality but also length of hospital stay (*P* = 0.011), care intensity, institutionalization, re-hospitalization, and access to homecare services [29, 30]. In an international multicenter cohort of 1140 hospitalized older patients, patients in group MPI-2 (OR 3.32 (1.79–6.17), *P* < 0.001) and the MPI-3 group (OR 10.72 (5.70–20.18), *P* < 0.0001) were at higher risk of overall mortality; compared to those of the lower risk group at admission [30]. This index may be used as a decision-making tree for cancer management, so as to select older patients with lower mortality risk for the same standard treatment as younger counterparts, those who could benefit from adapted care, or an exclusively supportive strategy in patients with limited life expectancy. This classification in three groups is comparable to the geriatric oncology algorithm of Balducci [4]. This algorithm defines three groups of patients (robust, vulnerable and frail) according to seven criteria: age, dependence measured by ADL and IADL, comorbidities with CIRS-CI, cognition evaluated with MMSE (mini-mental state examination) or delirium, depressive mood, urinary and fecal incontinence, and falls in the last 6 months. Risk of death increased steadily from the lowest to the highest category: compared to the fit group, the patients with a vulnerable profile had a two-fold mortality risk (HR 1.91 (0.95–3.85)) and a three-fold risk in the frail group (HR 2.94 (1.59–5.43), *P* < 0.001) [31]. More recent classifications were suggested to improve global management for such individuals, including nutrition data and cognitive assessment [25, 31].

Indeed, malnutrition is highly prevalent in geriatric oncology settings [32]. This geriatric syndrome is a well-known risk factor for early mortality. Our findings confirmed that one-year mortality is strongly associated with nutritional status and altered MNA in its short form. Some questions in this test were selected for the elaboration of the Geriatric-8 (G8) index, to screen for vulnerability in older patients with cancer, as recommended by the International Society for Geriatric Oncology (SIOG) [33, 34].

The findings of this study should be interpreted with caution. First, its design as an observational single-center study may limit the extrapolation of our results to a more general older population with cancer. Recruited patients in this cohort may not be representative, as cancer specialists may not refer all their patients to the geriatric oncology clinic, notably those screened as “not-vulnerable” in geriatric terms, as recommended by the SIOG and National Institute of Cancer in a two-step approach [33]. Cancer management of these patients may follow standard strategy, without geriatric expertise. After accounting for traditional risk factors, the magnitude of the improvement in risk prediction by the addition is small but significant. Moreover, our results are consistent with existing findings in geriatric oncology settings.

Our research on the predictive value of MPI for one-year mortality of older patients with cancer should serve as a foundation for future studies aiming to improve therapeutic strategies for these patients. A major part of this strategy involves personalized geriatric interventions, such as specific care monitoring by nurse and physical rehabilitation. It has shown benefits for elderly cancer patients, but so far, no study has demonstrated any impact on survival [35–37]. MPI appears to be a rapid assessment tool helping to optimize cancer care, guide patient-tailored interventions, and predict early mortality. These findings should pave the way for prospective interventional studies, taking account of MPI groups for decision-making about cancer treatments and follow-up.

## Conclusions

In addition to established risk factors, MPI improves risk prediction of 1-year mortality in older cancer patients. This practical prognostic tool may help to optimize management of these vulnerable individuals.

## Data Availability

The datasets used and/or analyzed during the current study are available from the corresponding author on reasonable request.

## Abbreviations

ADL: Activities of Daily Living
AIC: Akaike’s information criterion
CGA: Comprehensive Geriatric Assessment
CI: confidence interval
CIRS: Cumulative Illness Rating Scale
CIRS-CI: Cumulative Illness Rating Scale - comorbidity index
ESS: Exton-Smith Scale
G8: Geriatric-8
HR: Hazard ratio
IADL: Instrumental Activities of Daily Living
MNA-SF: Mini Nutritional Assessment-Short Form
MPI: Multidimensional Prognostic Index
SD: Standard deviation
SIOG: International society of geriatric oncology
SPMSQ: Short Portable Mental Status Questionnaire
